# Coadministration of the FNIII14 Peptide Synergistically Augments the Anti-Cancer Activity of Chemotherapeutic Drugs by Activating Pro-Apoptotic Bim

**DOI:** 10.1371/journal.pone.0162525

**Published:** 2016-09-13

**Authors:** Takuya Iyoda, Yumi Nagamine, Yoshitomi Nakane, Yuya Tokita, Shougo Akari, Kazuki Otsuka, Motomichi Fujita, Keisuke Itagaki, You-ichi Takizawa, Hiroaki Orita, Toshiyuki Owaki, Jyunichi Taira, Ryo Hayashi, Hiroaki Kodama, Fumio Fukai

**Affiliations:** 1 Department of Molecular Patho-Physiology, Faculty of Pharmaceutical Sciences, Tokyo University of Science, Noda, Chiba, Japan; 2 Translational Research Center, Research Institutes for Science and Technology, Tokyo University of Science, Noda, Chiba, Japan; 3 Department of Biochemistry, Faculty of Science and Engineering, Saga University, Saga, Saga, Japan; Wayne State University, UNITED STATES

## Abstract

The acquisition of drug resistance mediated by the interaction of tumor cells with the extracellular matrix (ECM), commonly referred to as cell adhesion-mediated drug resistance (CAM-DR), has been observed not only in hematopoietic tumor cells but also in solid tumor cells. We have previously demonstrated that a 22-mer peptide derived from fibronectin, FNIII14, can inhibit cell adhesion through the inactivation of β1 integrin; when coadministered with cytarabine, FNIII14 completely eradicates acute myelogenous leukemia by suppressing CAM-DR. In this study, we show that our FNIII14 peptide also enhances chemotherapy efficacy in solid tumors. Coadministration of FNIII14 synergistically enhances the cytotoxicity of doxorubicin and aclarubicin in mammary tumor and melanoma cells, respectively. The solid tumor cell chemosensitization induced by FNIII14 is dependent upon the upregulation and activation of the pro-apoptotic protein, Bim. Furthermore, the metastasis of tumor cells derived from ventrally transplanted mammary tumor grafts is suppressed by the coadministration of FNIII14 and doxorubicin. These results suggest that the coadministration of our FNIII14 peptide with chemotherapy could achieve efficient solid tumor eradication by increasing chemosensitivity and decreasing metastasis. The major causes of tumor recurrence are the existence of chemotherapy-resistant primary tumor cells and the establishment of secondary metastatic lesions. As such, coadministering FNIII14 with anti-cancer drugs could provide a promising new approach to improve the prognosis of patients with solid tumors.

## Introduction

Accompanied by the discovery of powerful anti-cancer drugs, the long-term survival of cancer patients has drastically improved in the last few decades. However, despite the sophisticated chemotherapeutic strategies which have been developed to date, tumor recurrence remains a major obstacle in the cure of various cancers. One of the causes of tumor recurrence is the persistence of cells which are insensitive to administered chemotherapeutic drugs. Given that tumor cells, especially solid tumor cells, frequently acquire drug resistant phenotypes during long-term chemotherapy, a means of restoring anti-cancer drug sensitivity is highly desirable and could help to achieve chemotherapeutic eradication of whole cancers.

Cell adhesion-mediated drug resistance (CAM-DR) is a type of drug resistance that has been described in hematological malignancies, and it has been shown that β1 integrin-mediated leukemic cell adhesion to fibronectin in the bone-marrow stroma plays a crucial role in the acquisition of this type of resistance [1–3]. Therefore, abrogation of β1 integrin signaling appears to be sufficient to overcome this form of drug resistance. We have previously found a biologically active peptide in fibronectin, FNIII14 [4, 5], and have shown that a synthetic peptide consisting of the FNIII14 site strongly alters the conformation of β1 integrin from its active to inactive form [6–9]. We have also demonstrated that CAM-DR in acute myelogenous leukemia (AML) cells is completely abrogated by the FNIII14 peptide; coadministration of FNIII14 with cytarabine efficiently eradicates leukemic cells from AML model mice, resulting in 100% survival during the observation period [2].

The acquisition of CAM-DR through β1 integrin ligation is not limited to hematological malignancies; it has also been observed in solid tumors. A survival advantage associated with β1 integrin-mediated cell adhesion and drug resistance has been reported in breast cancer and oral squamous cell carcinoma (OSCC) [10–12]. Furthermore, Nakagawa *et al*. recently reported that the CAM-DR observed in OSCC could be abrogated by our FNIII14 peptide [12]. However, to date, the mechanisms underlying the development of CAM-DR mediated by β1 integrin ligation have not been elucidated.

In the present study, using two different adherent tumor cell lines which originate from a mammary carcinoma and melanoma, we demonstrate that CAM-DR in solid tumor cells is abrogated by our FNIII14 peptide. Although Matsunaga *et al*. have reported that the abrogation of CAM-DR in AML cells is promoted through the downregulation of the anti-apoptotic protein Bcl-2, we found that the FNIII14 peptide enhanced expression and activation of the pro-apoptotic BH3-only Bcl-2 family protein, Bim, in solid tumor cells. This suggests that the molecular basis of FNIII14-mediated solid tumor cell chemosensitization is different to that observed in hematological malignancies. Furthermore, *in vivo* experiments show that coadministering FNIII14 with doxorubicin suppresses tumor metastasis without affecting the size of the primary tumor or subjects’ body weight. Taken together, the results presented suggest that FNIII14 coadministration is a promising strategy to sensitize tumor cells to chemotherapeutic drugs. By combining FNIII14 with chemotherapeutic drugs, tumor cell elimination and suppression of metastasis can be achieved, which could reduce the frequency of tumor recurrence.

## Materials and Methods

### Reagents

Human plasma fibronectin was purified as described previously [13]. Peptide FNIII14 corresponding to residues 1835–1855 of fibronectin [5] and its analogous inactive peptide FNIII14scr (amino acid scrambled peptide) were obtained from Sawady Technology. Vinblastine sulfate was purchased from Wako. Aclarubicin hydrochloide, doxorubicin hydrochloride, dacarbazine, z-VAD-fmk, and anti-actin antibody were purchased from Sigma. Antibodies against pan-Akt (sc-1618), Bax (sc-7480), Bcl-2 (sc-7832), and tubulin (sc-32293) were purchased from Santa Cruz. β1-integrin activating antibody (clone 9EG7) was from BD Pharmingen.

### Cell cultures

Mouse mammary tumor cell line 4T1 (kindly gifted from Dr. Kamiya at Josai international university) and MMT was cultured in complete RPMI medium supplemented with 10% heat-inactivated FBS and antibiotics. Mouse melanoma cell line B16BL6 (kindly gifted from Dr. Koshikawa at Yokohama city university) were cultured in complete DMEM/Ham’s F-12 (1:1) supplemented with 10% FBS and antibiotics. 4T1 and B16BL6 cells were maintained in monolayer and harvested with trypsin (0.05%)-EDTA (0.02%).

### WST assay

Cells (1.5 × 10^4^ cells/well) were seeded on a 96-well culture plate coated with 0.5 mg/mL of fibronectin in serum free medium. The relative number of viable cells was evaluated by the WST assay with a Cell Counting Kit-8 (DOJINDO) according to the manufacturer’s instructions. Absorbance at 450 nm was measured using a microplate reader (ARVO-MX 1420; PerkinElmer). The drug concentration producing 50% reduction in cell survival (IC_50_) was calculated for each drug from linear regression analysis of the linear portion of the survival curves.

### Combination index analysis

The combination index equation is based on the following multiple drug effect equation [14]: combination index = V_3_ / (V_1_ × V_2_) *100, V_1_; viability at indicated concentrations (C_1_) of anticancer drug, V_2_; viability at FNIII14, V_3_; viability at combination of anticancer drug (C_1_) and FNIII14. Combination index = 1, >1, or <1 is considered additive, antagonistic, or synergistic, respectively.

### Small RNA interference

The 21-mer duplex siRNA for Bim (target sequence of 5'- GACCGAGAAGGUAGACAAUUG -3'), and control siRNA (random-Bim; 5'- GGCUGUAACUUACGUGUACUU -3') were synthesized by Wakamori-syokai. Cells (1.0 × 10^5^ cells) were transfected with these siRNAs using Lipofectamin RNAiMAX (Life Technologies) according to the manufacturer’s instructions, and then seeded on 12-well plates. Twenty-four hours after transfection, cells were subjected to western-blot analysis or WST assay.

### Confocal microscopic analysis

Cells were seeded on cover-slips, which were coated with fibronectin (0.5 mg/ml), and cultured in complete growth medium. For mitochondrial staining, cells were loaded with 1 mM MitoTracker Red (Molecular Probes) at 37°C for 1 h. After fixation with cold methanol, cells were then permeabilized with 0.2% digitonin. After blocking step using 2% BSA, cells were stained with primary Abs followed by FITC- or Alexa633-conjugated secondary Ab. These cover-slips were then mounted on slide-glass and analyzed by confocal microscopy (Fluoview FV1000; Olympus).

### Sucrose gradient fractionation

Subcellular fractionation was performed as described previously [15]. Briefly, MMT cells, with or without FNIII14 and/or doxorubicin administration, were lysed in extraction buffer (1% Triton X-100, 50 mM PIPES, 50 mM HEPES, 2 mM MgCl_2_, 1 mM EDTA, 1 mM DTT) containing protease inhibitors. Lysates were taxol and apyrase for 15 min at 37°C. These samples were gently loaded onto an equal volume of 10% sucrose and centrifuged at 40,000 rpm in a swing rotor for 16 h at 4°C. Pellets were dissolved in Laemini buffer. Supernatants were concentrated by acetone precipitation, and then precipitants were dissolved in Laemini buffer. These samples were subjected to western blotting analysis.

### Immuno-precipitation

Cells were seeded on a 6-well plate and cultured for 4 h with BCG or reagents. The cells were then washed with PBS and lysed with 10 mM Tris-HCl (7.4) containing 150 mM NaCl, 1% NP-40, and inhibitors for proteinases and phosphatases. These lysates were incubated with anti-Bim Ab or anti-Bcl-2 Ab for 2 h at 4°C, following by incubation with Protein G-Sepharose beads (GE Healthcare) for 1 h at 4°C. Immuno-precipitants were then denatured with Laemmli’s buffer and subjected to western blotting as described above.

### *In vivo* model of spontaneous tumor metastasis

Female BALB/c mice aged 4 weeks were obtained from Sankyo Labo Service. The mice were initially maintained under standard conditions for 1 week with free access to food and water. After acclimatization, 3 x 10^6^ 4T1 cells were subcutaneously injected into dorsal flank of 5 weeks old mice (n = 3) in 200 mL PBS. After 11 days, the mice were euthanized by exsanguination under isoflurane anesthesia, and the primary tumor lesion was surgically excised and minced. Then, 0.6 mg of the primary tumor was transplanted into the ventral flank of another 5-week-old mouse. There were 16 mice that received transplanted tumors, and these were randomly divided into four equal groups: control, doxorubicin treatment, FNIII14 treatment, and doxorubicin+FNIII14 treatment. After transplantation, mice were intra-peritoneally injected with 200 mg/mouse FNIII14 in PBS on days 0 to 10. On day 4, 200 mg/mouse doxorubicin was also injected intra-peritoneally. The mice were sacrificed, as described above, 12 days after tumor transplantation. The lung, liver, and spleen were then collected and weighed. The organs were then formalin-fixed, paraffin-embedded, and tissue sections were cut and stained with hematoxylin/eosin. All of the animal procedures were approved by the Institutional Animal Care and Use Committee (IACUC) of Tokyo University of Science.

### Statistical analysis

Results are expressed as means ± standard deviation. Differences between experimental groups were analyzed using two-way factorial ANOVA and a post-hoc test. When a *p*-value was less than 0.05, the difference was considered statistically significant.

## Results

### The cytotoxicity of anti-cancer drugs is potentiated by the FNIII14 peptide in mammary tumor and melanoma cells

**Fig 1:**
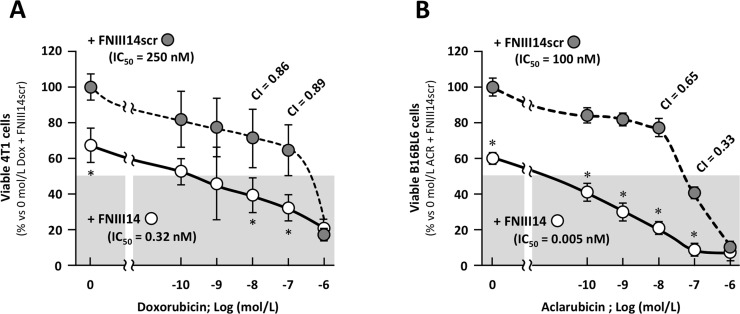
Coadministration of peptide FNIII14 synergistically increased susceptibility of tumor cells to chemotherapeutic drugs. (A) Mammary tumor cell line 4T1 was seeded on fibronectin-coated plated. Then these cells were treated with peptide FNIII14 and serial dose of doxorubicin (DOX). (B) Melanoma B16BL6 was seeded on fibronectin-coated plated. Then, these cells were treated with peptide FNIII14 and serial dose of aclarubicin (ACR). Twenty-four hours later, the number of viable cell was estinmated by WST assay. Data were shown as means ± S.D. *; *p*<0.05 vs the cells cotreated with FNIII14scr and anticancer drug. Combination index (CI) and IC_50_ values was calculated as described in materials and methods.

To determine whether the viability of solid tumor cells is affected by β1 integrin-derived signaling, the β1 integrin inactivating peptide, FNIII14, was coadministered with anti-cancer drugs in two solid tumor cell lines: 4T1 and B16BL6. These cells are derived from a mouse mammary tumor and melanoma, respectively. As shown in Fig 1A, a dose-dependent decrease in viable 4T1 cells was observed with doxorubicin treatment (closed gray circles), and similar results were obtained in B16BL6 cells treated with aclarubicin (Fig 1B, closed gray circles). As shown in S1A and S1E Fig, administration of the FNIII14 peptide to the solid tumor cell lines reduced their adhesion to fibronectin through inactivation of β1 integrin. When the cell lines were concomitantly treated with anti-adhesive FNIII14 peptide and anti-cancer drugs, the viability was dramatically decreased compared with cells treated with anti-cancer drugs and a control scramble FNIII14 (FNIII14scr) peptide, which has no effect on β1-integrin activation (Fig 1A and 1B, open circles vs. closed gray circles). These results suggest that tumor cell death is enhanced when the FNIII14 peptide is simultaneously administered with either doxorubicin or aclarubicin.

Of note, a decrease in the number of viable cells was also observed with administration of the peptide in the absence of anti-cancer drug treatment (Fig 1), and the adhesion of both cell lines to a fibronectin substrate was also suppressed by administration of the FNIII14 peptide alone (S1A and S1E Fig). The cytotoxic effect of the FNIII14 peptide observed in 4T1 and B16BL6 cells in the absence of anti-cancer drug administration was abrogated by the β1-integrin activating antibody 9EG7 (S1B and S1F Fig). In our experiments, the decrease in 4T1 cell viability that was induced by FNIII14 was abrogated by the broad caspase inhibitor Z-VAD-fmk (S1D Fig). Furthermore, pro-apoptotic caspase-3 activation was observed in the MMT mouse mammary tumor cell line (S1C Fig). These results indicate that the anti-adhesive FNIII14 peptide could induce apoptotic cell death in mammary tumor cells. Given these observations, we proceeded to determine whether the decrease in viability observed with coadministration of anti-cancer drugs with FNIII14 was due to cumulative FNIII14-induced anoikis and anti-cancer drug-induced apoptosis, or whether the effect of the two treatments was synergistic. To determine whether the effect of FNIII14 on viability is additive or synergistic to anti-cancer drug treatment alone, the combination index (CI: CI < 1.0 indicating a synergistic effect; CI = 1.0 indicating an additive effect; and CI > 1.0 indicating an antagonistic effect) value was calculated [14]. In FNIII14-treated 4T1 cells, the CI value was 0.86 at 10^−7^ M doxorubicin and 0.89 at 10^−8^ M doxorubicin; in FNIII14-treated B16BL6 cells, the CI values at 10^−7^ M and 10^−8^ M aclarubicin were 0.33 and 0.65, respectively. These data indicate that FNIII14 synergistically enhances the cytotoxic effect of anti-cancer drugs in solid tumor cell lines. To validate the FNIII14-mediated enhanced anti-cancer drug cytotoxicity, we also calculated the concentration of anti-cancer drug required to inhibit cell viability by half in the presence or absence of FNIII14 (IC_50_). The IC_50_ of doxorubicin in FNIII14-treated 4T1 cells was 0.32 nM, and 250 nM in control FNIII14scr-treated cells. When cells were treated only with doxorubicin, almost the same doxorubicin IC_50_ (210 nM) was obtained as in doxorubicin+FNIII14-scr-treated cells. In B16BL6 cells, the IC_50_ of aclarubicin coadministered with FNIII14 or the control peptide FNIII14scr was 0.005 nM and 100 nM, respectively. We proceeded to calculate the IC_50_ of a number of other anti-cancer drugs in B16BL6 cells in the presence and absence of the FNIII14 peptide (Table 1). The IC_50_ values calculated suggest that the cytotoxicity of a range of anti-cancer drugs is enhanced in the presence of the FNIII14 peptide, indicating that the FNIII14 peptide might broadly increase the susceptibility of tumor cells to anti-cancer drugs.

**Table 1:** Effect of peptide FNIII14 on the cytotoxicity of various anti-cancer drugs.

***B16BL6 cells***	**IC**_**50**_ **(x 10**^**−9**^ **M)**	
	+ FNIII14scr	+ FNIII14
***[Anti-biotics]***		
**Actinomycin D**	**10**	**0.05**
**Mytomycin C**	**10**	**0.025**
**Bleomycin**	**10**	**0.02**
***[Alkaloid]***		
**Vinblastine**	**10**	**0.001**
**Vincristine**	**50**	**0.01**
**Vindesine**	**1000**	**0.1**
***[Anti-metabolite]***		
**6-Melcaptoprine**	**1000**	**5**
**5-Fluorouracil**	**10**	**0.05**
***[Alkylating agent]***		
**Decarbazine**	**100**	**1**

Concentration of anti-cancer drug required to inhibit the cell viability by half in the presence or absence of FNIII14 (IC50) was calculated.

### Bim-mediated pro-apoptotic signaling underlies the synergistic enhancement of anti-cancer drug cytotoxicity by FNIII14

**Fig 2:**
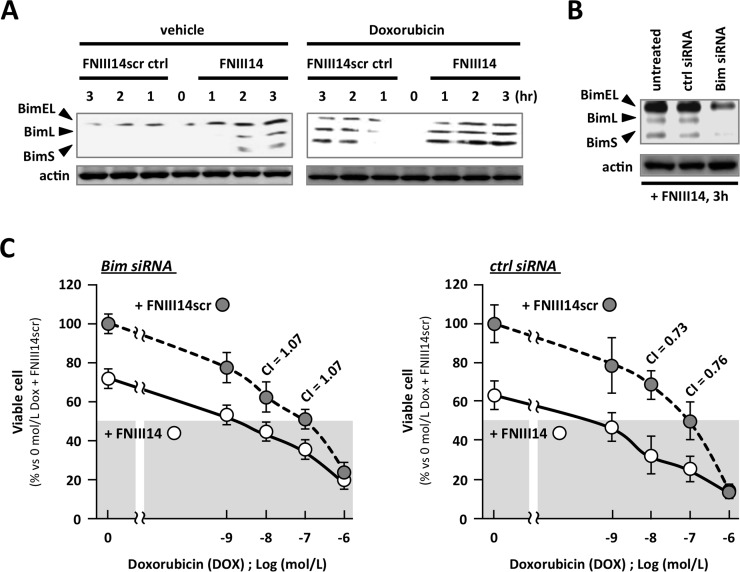
Implication of proapoptotic Bim protein in enhanced chemosensitivity induced by FNIII14 treatment. (A) 4T1 cells were seeded on fibronectin-coated plate. Then, these cells were stimulated with peptide FNIII14 and doxorubicin (DOX) for indicated hours. Whole cell lysates were subjected to western blotting analysis using anti-Bim Ab. (B) siRNA-mediated knockdown of Bim protein was attempted as described in materials and methods. Successful knockdown of Bim proteins was confirmed by western blotting analysis using anti-Bim Ab. (C) Serial dose of DOX was added to siRNA-transfected 4T1 cells combined with peptide FNIII14. Twenty-four hours later, the number of viable cell was estinmated by WST assay. Data were shown as means ± S.D. *; *p*<0.05 vs the cells treated with FNIII14scr and doxorubicin. Combination index (CI) was calculated as described in materials and methods.

In the last decade, the potential relationship between pro-apoptotic Bim and solid tumor cell chemosensitivity has been discussed [16–22]. We therefore proceeded to evaluate the expression of Bim in 4T1 cells treated with FNIII14 by western blotting. As shown in Fig 2A, BimEL levels were low in 4T1 cells treated with the control FNIII14scr peptide, and the alternative splice variants, BimL and BimS, were undetectable (Fig 2A, left side of left panel). All of these Bim proteins were upregulated in cells treated with the FNIII14 peptide alone (Fig 2A, right side of left panel) and in cells treated with doxorubicin alone (Fig 2A, left side of right panel). The coadministration of FNIII14 with doxorubicin resulted in a much greater increase in the levels of BimEL, BimL, and BimS (Fig 2A, right side of right panel).

Given that FNIII14 increased the expression of pro-apoptotic Bim, we proceeded to examine whether FNIII14-mediated upregulation of Bim was responsible for the enhanced tumor cell chemosensitivity. Initially, we established 4T1 cells with low levels of Bim expression by siRNA-mediated knockdown. As shown in Fig 2B, transfection of Bim siRNA into 4T1 cells greatly reduced the levels of the endogenous Bim variants, but the control siRNA oligonucleotides had no effect. When Bim-siRNA-transfected cells were cotreated with doxorubicin and FNIII14, the CI value was 1.07 at 10^−7^ M and 10^−8^ M doxorubicin; in control-siRNA-transfected cells, the CI value was 0.76 at 10^−7^ M doxorubicin and 0.73 at 10^−8^ M doxorubicin. Thus, the synergistic effect of FNIII14 on chemotherapeutic drug cytotoxicity was completely abrogated by downregulation of Bim. As such, FNIII14-induced upregulation of Bim could be responsible for the synergistic enhancement of drug-induced cell death by FNIII14.

**Fig 3:**
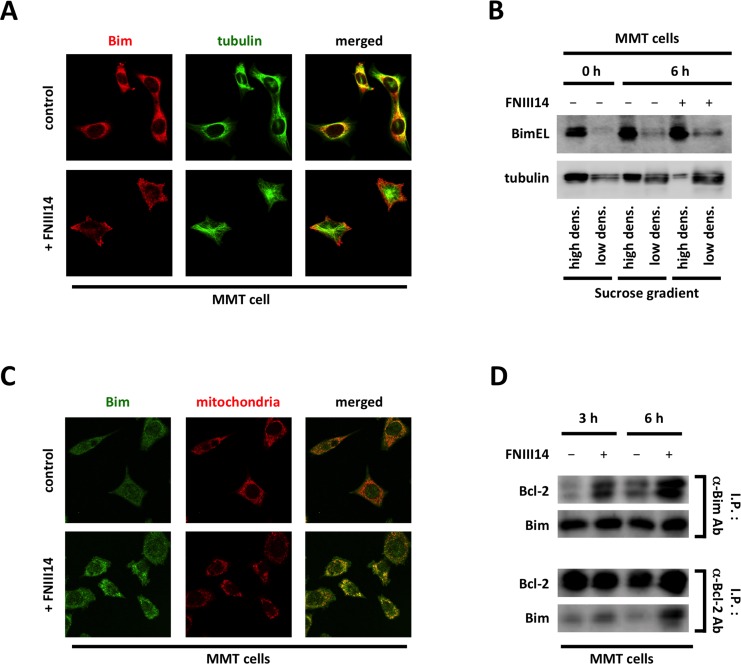
Activation of Bim signaling pathway in mammary tumor cells with FNIII14 treatment. (A) MMT cells were seeded on fibronectin-coated cover-slips and stimulated with peptide FNIII14. Then, immunofluoro-staining, using anti-Bim (red) and anti-tubulin (green) Abs, was performed and these samples were analyzed by confocal microscopy. Representative images from three independent experiments are shown. (B) Whole cell lysate of MMT cells with FNIII14 treatment was divided into a high-density and a low-density fraction by sucrose gradient centrifugation, and both fractioned samples were subjected to western blotting analysis with anti-Bim Ab and anti-tubulin Ab. (C) MMT cells were seeded on fibronectin-coated cover-slips and stimulated with peptide FNIII14 for 18 hrs. Then, immunofluoro-staining, using anti-Bim Ab (green) and mito-tracker (red), was performed and these samples were analyzed by confocal microscopy. Representative images from three independent experiments are shown. (D) Bcl-2 binding proteins and Bim binding proteins were immunoprecipitated from whole cell lysate of FNIII14-treated MMT cells using anti-Bcl-2 Ab and anti-Bim Ab, Precipitated proteins were subjected to western blotting analysis using anti-Bim Ab and anti-Bcl-2 Ab,

Bim-mediated pro-apoptotic signaling is regulated not only at the level of expression, but also by changes in Bim subcellular localization. Pro-apoptotic Bim is inactive in cells while adhered to the extracellular matrix (ECM) substrate. Bim proteins are sequestered through binding to the microtubules distributed throughout the cytoplasm. When the Bim proteins are released from the microtubules, they translocate to the mitochondrial membrane where they capture anti-apoptotic proteins such as Bcl-2 and Bcl-xL, resulting in the induction of apoptosis. To investigate whether the FNIII14 peptide influences the subcellular localization of Bim, we used confocal microscopy and anti-Bim (red) and anti-tubulin (green) antibodies to investigate Bim localization in 4T1 cells. As shown in the left panel of S3A Fig, a merged yellow image, which indicates colocalization of Bim and tubulin, was observed in untreated cells. However, an entirely different pattern of localization (green and red images) was observed in FNIII14-treated cells. Similar results were obtained in the mouse mammary tumor MMT cell lines (Figs 3A and S3B), suggesting that FNIII14 induces the release of Bim from microtubules. Moreover, in MMT cells, Bim release from microtubules was confirmed in a sucrose density gradient fractionation study. After centrifugation, whole MMT cell lysates were divided into a high-density fraction, which includes tubulin polymers, and a low-density fraction, which includes tubulin monomers. The fractions were then subjected to western blotting. In the low-density fraction, the level of BimEL was increased when cells were treated with FNIII14 (Fig 3B, upper panel), concomitant with increased tubulin depolymerization (Fig 3B, lower panel). We proceeded to perform confocal microscopy to visualize the colocalization of Bim with the mitochondria using an anti-Bim antibody (green) and mitotracker (red). As shown in Fig 3C, a merged yellow image, indicating the colocalization of Bim with the mitochondria, was observed in cells treated with FNIII14 (lower panel), but a different localization pattern (green and red image; upper panel) was observed in control cells. Furthermore, an immuneprecipitation study indicated that with FNIII14 treatment, Bim forms a complex with the anti-apoptotic protein Bcl-2, which is expressed on the mitochondrial membrane (Fig 3D). These results indicate that the enhanced chemosensitivity observed in tumor cells with FNIII14 treatment is caused by the activation of pro-apoptotic Bim signaling through modulation of its expression and subcellular localization.

Drug-resistant cells have frequently been shown to have elevated efflux capacity compared with chemosensitive cells [23–25], and so we investigated whether FNIII14-mediated chemosensitization is achieved through the suppression of drug efflux. However, an analysis of intracellular drug accumulation, performed using flow cytometry, indicated that there was no difference in the level of intracellular doxorubicin or aclarubicin in FNIII14 peptide and control FNIII14scr-treated cells (S2 Fig). This suggests that the enhanced chemosensitivity observed in FNIII14-treated cells does not result from alterations in drug influx/efflux.

### Coadministration of FNIII14 with doxorubicin inhibits the metastasis of 4T1 cells

**Fig 4:**
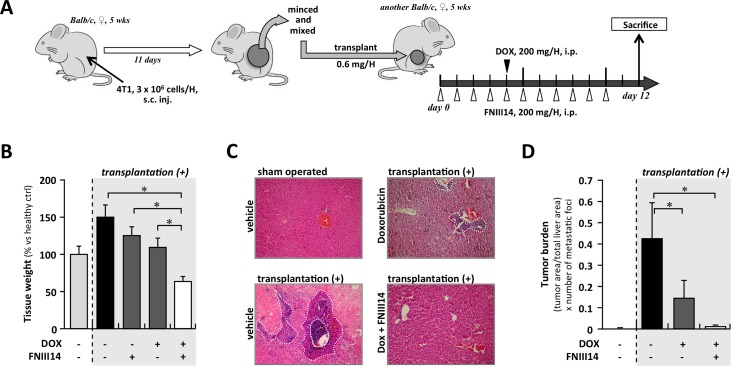
Combination therapy using FNIII14 and doxorubicin in mouse mammary tumor metastasis model. (A) The schematic illustration of experimental schedule. (B) Liver weight at day 12 after tumor graft implantation was shown in bar graph. Data were shown as means ± S.D. *; *p*<0.05 vs Dox(-)/FNIII14(-) animals. (C) Representative image of paraffin-embedded tissue section with hematoxylin-eosin staining. Metastatic tumor lesion was circled by broken white line. (D) Tumor burden was calculated as [(tumor area / total tissue area) / number of metastatic foci].

Finally, we evaluated the clinical potential of the FNIII14 peptide *in vivo*. Since we had determined that the anti-tumor effect of FNIII14 was derived from activation of Bim signaling, and accumulating evidence suggests that the suppression of Bim in tumor cells plays a significant role in tumor metastasis rather than in primary tumorigenesis [26], we focused our studies on the effect of FNIII14 on tumor metastasis. To prepare our tumor metastasis model, 4T1 cells were subcutaneously injected into the dorsal flank of Balb/c mice (n = 3). After 11 days, the established tumors were excised, minced, and 0.6 mg of tumor was transplanted into the ventral flank of healthy Balb/c mice (n = 16). These mice were randomly divided into 4 groups that were administered doxorubicin, FNIII14, doxorubicin+FNIII14, or vehicle, as shown in Fig 4A. After 12 days, the frequency of metastasis was estimated by weighing tissues. As shown in Fig 4B, an approximate 1.5-fold increase in the liver wet weight was observed in mice after tumor transplantation (black bar), although no differences in the body weight or primary tumor size were observed (S4A and S4B Fig). Histological analyses indicated that the increase in liver weight was due to the establishment of metastatic lesions (Fig 4C, lower left, and Fig 4D black bar). Mice treated with doxorubicin alone or the FNIII14 peptide alone showed a slight but significant suppression of the liver weight increase (Fig 4B, dark gray bars). However, a remarkable suppression of metastasis-induced liver weight gain was observed in mice concomitantly administered doxorubicin and FNIII14 (Fig 4B, open bar). Histology also indicated that mice coadministered doxorubicin and FNIII14 showed reduced levels of metastatic lesion establishment (Fig 4C, lower right). As shown in Fig 4D, the metastatic tumor burden in the livers of FNIII14+doxorubicin treated mice was also significantly reduced. No abnormal features, such as inflammatory or necrotic signs, were observed in the liver tissue sections derived from FNIII14+doxorubicin treated mice, although the liver weight in this group was less than that in normal healthy group (Fig 4B). In mammary tumor transplanted mice, an increase in lung and spleen tissue weight was also observed. This was significantly suppressed when mice were coadministered doxorubicin and FNIII14 (S4C and S4E Fig); the lung metastatic tumor burden in FNIII14 and doxorubicin co-treated mice was significantly reduced (S4D Fig). Thus, our results suggest that the FNIII14 peptide, in combination with chemotherapeutic drugs, has much therapeutic potential as an inhibitor of tumor metastasis.

## Discussion

In this study, we have provided two lines of evidence to show that coadministration of our anti-adhesive FNIII14 peptide with chemotherapy could provide a new and efficacious approach for the clinical treatment of solid tumors. Firstly, we have shown *in vitro* that the efficacy of a number of anti-cancer drugs is synergistically enhanced in solid tumor cells by concomitant treatment with FNIII14. Secondly, we have demonstrated *in vivo* that the FNIII14 peptide can suppress the formation of metastatic lesions in mice with mammary tumor transplants. Although tumor recurrence remains a major obstacle in the elimination of solid cancers, our observations suggest that the FNIII14 peptide has the potential to overcome this through its ability to enhance the efficacy of chemotherapeutic drugs.

The majority of anti-cancer chemotherapeutic drugs exert their cytotoxic effects by stimulating intrinsic pro-apoptotic signaling, which is activated through the perturbation of mitochondrial homeostasis. Mitochondrial apoptosis is essentially regulated by a complex interplay of pro- and anti-apoptotic Bcl-2 family proteins. Consistent with this theory, it has been reported that the acquisition of CAM-DR in AML cells is due to upregulation of the anti-apoptotic Bcl-2 protein induced by β1 integrin-mediated cell adhesion [1]. We have shown that our FNIII14 peptide can abrogate CAM-DR through its ability to inactivate β1 integrin [2]. Although the anti-cancer drug sensitivity of solid tumor cells was increased by FNIII14 in the present study (Fig 1 and Table 1), as is the case in AML cells, the enhanced chemosensitivity observed was accompanied by activation of pro-apoptotic Bim signaling (Figs 2, 3 and S3). Here, we have showed that the synergistic enhancement of doxorubicin cytotoxicity by FNIII14 is completely abrogated by Bim knockdown. These facts suggest that the mechanism underlying CAM-DR acquisition is different in adherent solid tumor cells and non-adherent hematological tumor cells. Supporting this hypothesis, it has previously been reported that doxorubicin exerts its effects through the Bim signaling pathway in various solid tumor cells [16, 21, 22]. Furthermore, a correlation between drug resistance and Bim activation has also been reported for other chemotherapeutic drugs in solid tumor cells [17–20]. Considering that Bim expression is frequently suppressed in solid tumor cells, FNIII14-mediated activation of Bim signaling, which should make solid tumor cells more sensitive to chemotherapeutic drugs, could be beneficial for anti-solid tumor drug therapy.

The upregulation of multidrug-resistance (MDR)-1 is a well-described principal mechanism underlying the acquisition of drug resistance [23–25]. Since the induction of MDR-1 expression by EGF has been reported in breast cancer cells [27], and EGFR expression is induced as a consequence of β1 integrin ligation [28], we hypothesized that FNIII14-mediated inactivation of β1 integrin might impact cellular drug efflux capacity. However, flow cytometry performed as part of this study indicated that there was no difference in the intracellular accumulation of doxorubicin in FNIII14-treated and control peptide-treated 4T1 cells (S2A Fig), and similar results were obtained with regard to intracellular vinblastine levels in B16BL6 melanoma cells (S2B Fig). This suggests that the increased cytotoxicity of chemotherapeutic drugs observed in cells treated with FNIII14 was not due to increased intracellular drug accumulation; FNIII14 might enhance the cytotoxicity of chemotherapeutic drugs by modulating the sensitivity of tumor cells to pro-apoptotic stimuli. If this is the case, then FNIII14 might also be applicable to other types of drug-free cancer therapy, such as radiation therapy. Indeed, it has been reported that the efficacy of ionizing radiation in human breast cancer xenografts is enhanced by antibody-mediated β1 integrin inhibition [29]. Furthermore, an essential role for β1 integrin-derived signaling has also been demonstrated in the acquisition of breast cancer invasiveness during radiotherapy [30]. In the present study, we have only tested the effect of FNIII14 on chemotherapeutic drug efficacy. However, we anticipate that administration of FNIII14 might also enhance the efficacy of other therapeutic approaches used in clinical oncology, such as radiation therapy.

Although we have demonstrated that the cytotoxic effect of anti-cancer drugs is synergistically enhanced by FNIII14 (Fig 1), the viability of mammary tumor and melanoma cells was also decreased in the presence of FNIII14 alone. In normal healthy tissue, the intracellular signaling which is activated by cell adhesion receptor integrins plays a key role in the regulation of growth and cell survival. In normal solid tissue, cells undergo apoptotic cell death, sometimes referred to as anoikis, when they lose adhesion to the appropriate ECM substrate. In contrast, it is well accepted that resistance to anoikis exists in malignant solid tumor cells; metastatic potential is obtained through the acquisition of anokis resistance which enables metastatic cells to grow and survive in an inappropriate ECM environment. Thus, our observation that the viability of solid tumor cells is decreased by FNIII14-mediated inactivation of β1 integrin, appears to be incongruous with our general understanding of malignant solid tumor cells. However, supporting our observations, Lee *et al*. have previously reported that anoikis resistance in prostate cancer cells is abolished by β1 integrin knockdown [31], and Morozevich *et al*. have reported that anoikis resistance in MCF-7 mammary tumor cells is accompanied by enhanced α5 β1 integrin expression [32]. Sanches-Ruderisch *et al*. have also shown that anoikis resistance in hepatocellular carcinoma HepG2 cells is abrogated by endogenous lectin galectin-1, which can interact with unligated α5 β1 integrins [33]. Moreover, consistent with our observation that FNIII14 enhances Bim expression and activation, the suppression of Bim signaling been reported to be involved in the acquisition of anoikis resistance. Fukazawa *et al*. have reported that the in human breast cancer, anoikis resistance is maintained through the low levels of Bim expression which result from ERK-mediated Bim degradation [34]. Buchheit *et al*. have also shown that ERK-mediated Bim sequestration is implicated in the evasion of anoikis in inflammatory breast cancer cells [35]. It has been well documented that the expression of Bim is regulated by forkhead transcription factors, which bind to the Bim gene promoter region. This binding is suppressed by Akt activation and the well described activation of MEK/ERK and PI3K/Akt through FAK. Taken together, these observations suggest that our FNIII14 peptide also has the potentially be used clinically to abrogate the anoikis resistance observed in malignant solid tumor cells.

Besides demonstrating that Bim signaling plays a key role in FNIII14-mediated tumor cell chemosensitization, and that FNIII14 has clear potential for the suppression of tumor metastasis. Although we have not elucidated the mechanism underlying the suppression of tumor metastasis, it is possible that the adhesion of circulating tumor cells, derived from the transplanted graft, was suppressed by FNIII14-mediated inactivation of β1 integrin, thereby reducing their capacity to establish metastases in distant tissues. However, a key role for intracellular HDAC6 and MT1-MMP transport in tumor cell metastasis has also been recently reported [36, 37]. These two molecules are loaded to the dynein motor complex and transported along the microtubule network. Interestingly, pro-apoptotic Bim is sequestered through loading to the dynein motor complex [15]. In this study, we observed that Bim expression in solid tumor cells was significantly enhanced by FNIII14 treatment, suggesting that the level of HDAC6 and MT1-MMP loaded to the dynein motor complex could possibly be reduced. Furthermore, the dynein motor complex would also be disrupted by the activation of pro-apoptotic Bim signaling [38]. As such, it is possible that the anti-metastatic effect of FNIII14 is due, at least in part, to the defective transport of molecules required for tumor metastasis, although further studies are required.

In this study, we have demonstrated that the β1 integrin inactivating peptide, FNIII14, can abrogate drug resistance and anoikis resistance in solid tumor cells, and we have shown that Bim plays a fundamental role in this. We have also demonstrated that FNIII14 administration can significantly suppress tumor metastasis *in vivo*. Although various β1 integrin heterodimers have been reported to transduce survival signals in solid tumor cells, we have previously shown that FNIII14 can inhibit β1 integrin regardless of the associated α-subunit [6–9]. Moreover, we have also previously determined that the FNIII14 peptide exerts no myelosuppressive effects in mice treated with FNIII14 and Ara-C [2]. Although the liver weight of mice treated with doxorubicin+FNIII14 was lower than untreated healthy mice in this study, our histopathological analysis indicated no abnormal features. Furthermore, no significant differences were observed in the weight of other tested tissues, or body weight, of doxorubicin+FNIII14-treated and normal healthy mice.

Taken together, our results indicate that the efficacy of chemotherapeutic drugs can be improved by administering the FNIII14 peptide with anti-cancer drugs. The FNIII14 peptide has the potential to provide a promising new clinical approach to achieving complete cancer cell eradication using anti-cancer chemotherapies. Furthermore, since FNIII14 enhances the cytotoxicity of anti-cancer drugs synergistically, the coadministration of FNIII14 could enable the anti-cancer drug dose to be reduced. This could potentially reduce the burden of side effects associated with anti-cancer drugs, and also reduce the late effects of anti-cancer drugs, such as cardiopulmonary toxicity and endocrinopathy in cancer survivors. Thus, although further studies are required, in particular to elucidate the reason for the reduced liver weight of FNIII14+doxorubicin-treated mice, FNIII14 may be able to contribute to maintaining a high quality of life in patients following chemotherapy; FNIII14 has the potential to achieve complete cancer eradication and reduce the dose of chemotherapeutics required.

## Supporting Information

S1 Text Materials and Methods section for S1 Fig and S2 Fig. (PDF)

S1 Fig Adhesion and survival of tumor cells treated with anti-adhesive peptide FNIII14. 4T1 (A, B, D) or B16BL6 (E, F) cells were seeded on fibronectin-coated plate with/without peptide FNIII14, β1 integrin activating antibody 9EG7, and caspase inhibitor Z-VAD. One and a half hours later, number of adhered cells (both spread and attached cells) in random 5 fields was counted after crystal violet staining (A, E). Twenty-four hours later, the number of viable cell was estimated by WST assay (B, D, F). Data were shown as means ± S.D. *; p<0.05 vs FNIII14(-)/9EG7(-) cells. (C) Cleavage of caspase-3 in MMT cells with FNIII14 treatment was evaluated by western blotting.
(PDF)

S2 Fig Effect of FNIII14 on intracellular accumulation of chemotherapeutic drug. (A) Doxorubicin (DOX)-treated 4T1 cells with/without FNIII14 administration was harvested and intracellular fluorescence was measured by flowcytometer. (B) FITC-conjugated vinblastine (VBL)-treated B16BL6 cells with/without FNIII14 administration was harvested and intracellular fluorescence was measured by flowcytometer.
(PDF)

S3 Fig Activation of Bim signaling pathway in mammary 1 tumor cells with FNIII14 treatment. (A) 4T1 cells, stimulated with peptide FNIII14, was stained using anti-Bim (red) and anti-tubulin (green) Abs. Representative images of confocal microscopic analysis from three independent experiments are shown. (B) Traces of fluorescence intensity spatial profile through the white broken line shown in confocal images (upper left of each histogram). White arrowhead displays a positive correlative colocalization of Bim and tubulin, or Bim and mitochondria, while white arrow indicates a absence of correlative colocalization.
(PDF)

S4 Fig Combination therapy using FNIII14 and doxorubicin in mouse mammary tumor metastasis model. Body weight (A), implanted primary tumor size (B), and the weight of lung (C) and spleen (D) at day 12 after tumor graft implantation was shown in bar graph. Data were shown as means ± S.D. *; p<0.05 vs Dox(-)/FNIII14(-) animals.
(PDF)

## References

[1] Matsunaga T, Takemoto N, Sato T, Takimoto R, Tanaka I, Fujimi A, et al Interaction between leukemic-cell VLA-4 and stromal fibronectin is a decisive factor for minimal residual disease of acute myelogenous leukemia. Nat Med. 2003; 9: 1158–1165. 12897778 10.1038/nm909

[2] Matsunaga T, Fukai F, Miura S, Nakane Y, Owaki T, Kodama H, et al Combination therapy of an anticancer drug with the FNIII14 peptide of fibronectin effectively overcomes cell adhesion-mediated drug resistance of acute myelogenous leukemia. Leukemia, 2008; 22: 353–360. 17972943 10.1038/sj.leu.2405017

[3] Canpinar H, Esendagli G, Kansu E, Gunel-Ozcan A, Guc D. Adhesion of beta1 integrin to fibronectin regulates CAM-DR phenotype via p21waf1/cip1 in HL60 acute myeloid leukemia (AML) cells. Turk J Med Sci. 2008; 38: 97–104.

[4] Fukai F, Takahashi H, Habu Y, Kubushiro N, Katayama T. Fibronectin harbors anticell adhesive activity. Biochem Biophys Res Commun. 1996; 220: 394–398. 8645316 10.1006/bbrc.1996.0416

[5] Fukai F, Hasebe S, Ueki M, Mutoh M, Ohgi C, Takahashi H, et al Identification of the antiadhesive site buried within the heparin-binding domain of fibronectin. J Biochem (Tokyo). 1997; 121: 189–192. 9089388

[6] Fukai F, Mashimo M, Akiyama K, Goto T, Tanuma S, Katayama T. Modulation of apoptotic cell death by extracellular matrix proteins and a fibronectin-derived anti-adhesive peptide. Exp Cell Res. 1998; 242: 92–99. 9665806 10.1006/excr.1998.4076

[7] Fukai F, Kamiya S, Ohwaki T, Goto S, Akiyama K, Goto T, et al The fibronectin derived anti-adhesive peptide FNIII14 suppresses adhesion and apoptosis of leukemic cell lines through downregulation of protein-tyrosine phosphorylation. Cell Mol Biol. 2000; 46: 145–152. 10726980

[8] Kato R, Ishikawa T, Kamiya S, Oguma F, Ueki K, Goto S, et al A new type anti-metastatic peptide derived from fibronectin. Clin Cancer Res. 2002; 8: 2455–2462. 12114453

[9] Kamiya S, Kato R, Wakabayashi M, Tohyama T, Enami I, Ueki M, et al Fibronectin peptides derived from two distinct regions stimulate adipocyte differentiation by preventing fibronectin matrix assembly. Biochemistry. 2992; 41: 3270–3277. 11863466 10.1021/bi015660a

[10] Nista A, Leonetti C, Bernardini G, Mattioni M, Santoni A. Functional role of a4b1 and a5b1 integrin fibronrctin receptors expressed on Adriamycin-resistant MCF-7 human mammary carcinoma cells. Int J Cancer. 1997; 72: 133–141. 9212234 10.1002/(sici)1097-0215(19970703)72:1<133::aid-ijc19>3.0.co;2-k

[11] Aoudjit F, Vuori K. Integrin signaling inhibits paclitaxel-induced apoptosis in breast cancer cells. Oncogene. 2001; 20: 4995–5004. 11526484 10.1038/sj.onc.1204554

[12] Nakagawa Y, Nakayama H, Nagata M, Yoshida R, Kawahara K, Hirosue A, et al Overexpression of fibronectin confers cell adhesion-mediated drug resistance (CAM-DR) against 5-FU in oral squamous cell carcinoma cells. Int J Oncol. 2014; 44: 1376–1384. 10.3892/ijo.2014.2265 24452447

[13] Miekka SI, Ingham KC, Menache D. Rapid methods for isolation of human plasma fibronectin. Thromb Res. 1982; 27: 1–14. 6812233 10.1016/0049-3848(82)90272-9

[14] Drewinko B, Green C, Loo TL. Combination chemotherary in vitro with cis-dichloro- diammineplatium(II). Cancer Treat Rep. 1976; 60: 1619–1625. 1021234

[15] Puthalakath H, Huang DCS, O’Reilly LA, King SM, Strasser A. The proapoptotic activity of the Bcl-2 family member Bim is regulated by interaction with the dynein motor complex. Mol Cell. 199; 3: 287–296. 10198631 10.1016/s1097-2765(00)80456-6

[16] Li S, Zhou Y, Wang R, Zhang H, Dong Y, Ip C. Selenium sensitizes MCF-7 breast cancer cells to doxorubicin-induced apoptosis through modulation of phosphor-Akt and its downstream substrates. Mol Cancer Ther. 2007; 6: 1031–1038. 17339365 10.1158/1535-7163.MCT-06-0643

[17] Yan H, Liu W, Sun W, Wu J, Ji M, Wang Q, et al miR-17-5p inhibitor enhances chemosensitivity to gemcitabine via upregulating Bim expression in pancreatic cancer cells. Dig Dis Sci. 2012; 57: 3160–3167. 10.1007/s10620-012-2400-4 23001407

[18] Savry A, Carre M, Berges R, Rovini A, Pobel I, Chacon C, et al Bcl-2-enhanced efficacy of microtubule-targeting chemotherapy through Bim overexpression: implication for cancer treatment. Neoplasia. 2013; 15: 49–60. 23358890 10.1593/neo.121074 PMC3556938

[19] Nakagawa T, Takeuchi S, Yamada T, Ebi H, Sano T, Nanjo S, et al EGFR-TK1 resistance due to Bim polymorphism can be circumvented in combination with HDAC inhibition. Cancer Res. 2013; 73: 2428–2434. 10.1158/0008-5472.CAN-12-3479 23382048

[20] Li H, Zhou S, Li X, Wang D, Wang Y, Zhou X, et al Gefitinib-resistance is related to Bim expression in non-small cell lung cancer cell lines. Cancer Biother Radiopharm. 2013; 28: 115–123. 10.1089/cbr.2012.1268 23270470 PMC3589885

[21] Park HK, Lee JE, Lim J, J DE, Park SA, Suh PG, et al Combination treatment with doxorubicin and gamitrinib synergistically augments anti-cancer activity through enhanced activation of Bim. BMC Cancer. 2014; 14: 431–439. 10.1186/1471-2407-14-431 24927938 PMC4072609

[22] Yang MC, Lin RW, Huang SB, Huang SY, Chen WJ, Wang S, et al Bim directly antagonizes Bcl-xl in doxorubicin-induced prostate cancer cell apoptosis independently of p53. Cell Cycle. 2015 22; 00–00. 10.1080/15384101.2015.1127470 PMC4943702 26694174

[23] Hamada H, Tsuruo T. Functional role for the 170- to 180-kDa glycoprotein specific to drug-resistant tumor cells as revealed by monoclonal antibodies. Proc Natl Acad Sci USA. 1986; 83: 7785–7789. 2429319 10.1073/pnas.83.20.7785 PMC386806

[24] Hamada S, Tsuruo T. Purification of the 170- to 180-kilodalton membrane glycoprotein associated with multidrug resistance. J Biol Chem. 1988; 263: 1454–1458. 2891711

[25] Naito M, Hamada H, Tsuruo T. ATP/Mg^2+^-dependent binding of vincristine to plasma membrane of multidrug-resistant K562 cells. J Biol Chem. 1988; 263: 11887–11891. 3165378

[26] Akiyama T, Dass CR, Choong PF. Bim-targeted cancer therapy: A link between drug action and underlying molecular changes. Mole Cancer Ther. 2009; 8: 3137–3180. 10.1158/1535-7163.MCT-09-0685 19934277

[27] Garcia R, Franklin RA, McCubrey JA. EGF induces cell motility and multi-drug resistance gene expression in breast cancer cells. Cell Cycle. 2006; 5: 2820–2826. 17172846 10.4161/cc.5.23.3535

[28] Reginato MJ, Milles KR, Paulus JK, Lynch DK, Sgroi DC, Debnath J, et al Integrin and EGFR coordinately regulate the pro-apoptotic protein Bim to prevent anoikis. Nat Cell Biol. 2003; 5: 733–740. 12844146 10.1038/ncb1026

[29] Park CC, Zhang HJ, Yao ES, Park CJ, Bissell MJ. B1 integrin inhibition dramatically enhances radiotherapy efficacy in human breast cancer xenografts. Cancer Res. 2008; 68: 4398–4405. 10.1158/0008-5472.CAN-07-6390 18519702 PMC3719863

[30] Nam JM, Ahmad KM, Costes S, Zhang H, Onodera Y, Olshen AB, at al. β1-integrin via NF-αB signaling is essential for acquisition of invasiveness in a model of radiation treated in situ breast cancer. Breast Cancer Res. 2013; 15: 60–74. 10.1186/bcr3454 PMC3978561 23883667

[31] Lee YC, Jin JK, Cheng CJ, Huang CF, Song JH, Huang M, et al Targeting constitutively activated b1 integrins inhibits prostate cancer metastasis. Mol Cancer Res. 2013; 11: 405–417. 10.1158/1541-7786.MCR-12-0551 23339185 PMC3631285

[32] Morozevich GE, Kozlova NI, Preobrazhenskaya ME, Ushakova NA, Eltsov IA, Shtil AA, et al The role of b1 integrin subfamily in anchorage-dependent apoptosis of breast carcinoma cells differing in multidrug resistance. Biochemistry (Mosc). 2006; 71: 489–95. 16732726 10.1134/s000629790605004x

[33] Sanchez-Ruderisch H, Detjen KM, Welzel M, Andre S, Fischer C, Gabius HJ, et al Galectin-1 sensitizes carcinoma cells to anoikis via the fibronectin receptor a5b1-integrin. Cell Death Differ. 2011; 18: 806–816. 10.1038/cdd.2010.148 21113146 PMC3131929

[34] Fukazawa H, Noguchi K, Masui A, Murakami Y, Uehara Y. BimEL is an important determinant for induction of anoikis sensitivity by mitogen-activated protein/extracellular signal-regulated kinase kinase inhibitors. Mol Cancer Ther. 2004; 3: 1281–1288. 15486195

[35] Buchheit CL, Angorola BL, Steiner A, Weigel KJ, Schafer ZT. Anoikis evasion in inflammatory breast cancer cells is mediated by Bim-EL sequestration. Cell Death Differ. 2015; 22: 1275–1286. 10.1038/cdd.2014.209 25526094 PMC4495353

[36] Aidana-Masangkay GI, Sakamoto KM. The role of HDAC6 in cancer. J Biomed Biotechnol. 2011; 2011: 875824 10.1155/2011/875824 21076528 PMC2975074

[37] Marchesin V, Castro-Castro A, Lodillinsky C, Castagnino A, Cyrta J, Bonsang-Kitzis H, et al ARF6-JIP3/4 regulate endosomal tubules for MT1-MMP exocytosis in cancer invasion. J Cell Biol. 2015; 211: 339–358. 10.1083/jcb.201506002 26504170 PMC4621834

[38] Song C, Wen W, Rayala SK, Chen M, Ma J, Zhang M, et al Serine 88 phosphorylation of 8-kDa dynein light chain 1 is a molecular switch for its dimerization status and functions. J Biol Chem. 2008; 283, 4004–4013. 18084006 10.1074/jbc.M704512200

