# Supplementary material for: Coadministration of the FNIII14 Peptide Synergistically Augments the Anti-Cancer Activity of Chemotherapeutic Drugs by Activating Pro-Apoptotic Bim
Source: PLoS One. 2016 Sep 13;11(9):e0162525. doi: 10.1371/journal.pone.0162525 (PMC5021278; doi:10.1371/journal.pone.0162525)
Supplement: S1 Text — (PDF) [file pone.0162525.s001.pdf]

S1 Text

Materials and methods

Cell adhesion assay

Cells (1.5×10^4 cells/well) were seeded on a 96-well plate coated with fibronectin (0.5 mg/mL) in serum free medium, and then incubated at 37℃ for 1.5 h. Cells were then incubated with the indicated reagents at 37℃ for 1.5 h. After removing non-adhered cells by repeated washing, cells were fixed with 4% formaldehyde and stained with crystal violet. Cell adhesion was evaluated by the number of spread and attached cells.

Intracellular drug accumulation assay

Cells (5×10^5) were exposed to 10 nmol/L DOX or FITC-VBL with or without FNIII14 (100 mg/mL) for indicate time at 37°C. Intracellular accumulation of these drugs was evaluated by flow cytometer (FACS Aria, BD Biosciences). In this assay, a portion of cells was exposed to these drugs on ice to evaluate nonspecific binding of drugs to plasma membranes.
