# Supplementary material for: Coadministration of the FNIII14 Peptide Synergistically Augments the Anti-Cancer Activity of Chemotherapeutic Drugs by Activating Pro-Apoptotic Bim
Source: PLoS One. 2016 Sep 13;11(9):e0162525. doi: 10.1371/journal.pone.0162525 (PMC5021278; doi:10.1371/journal.pone.0162525)
Supplement: S1 Fig — 4T1 (A, B, D) or B16BL6 (E, F) cells were seeded on fibronectin-coated plate with/without peptide FNIII14, β1 integrin activating antibody 9EG7, and caspase inhibitor Z-VAD. One and a half hours later, number of adhered cells (both spread and attached cells) in random 5 fields was counted after crystal violet staining (A, E). Twenty-four hours later, the number of viable cell was estimated by WST assay (B, D, F). Data were shown as means ± S.D. *; p<0.05 vs FNIII14(-)/9EG7(-) cells. (C) Cleavage of caspase-3 in MMT cells with FNIII14 treatment was evaluated by western blotting. (PDF) [file pone.0162525.s002.pdf]

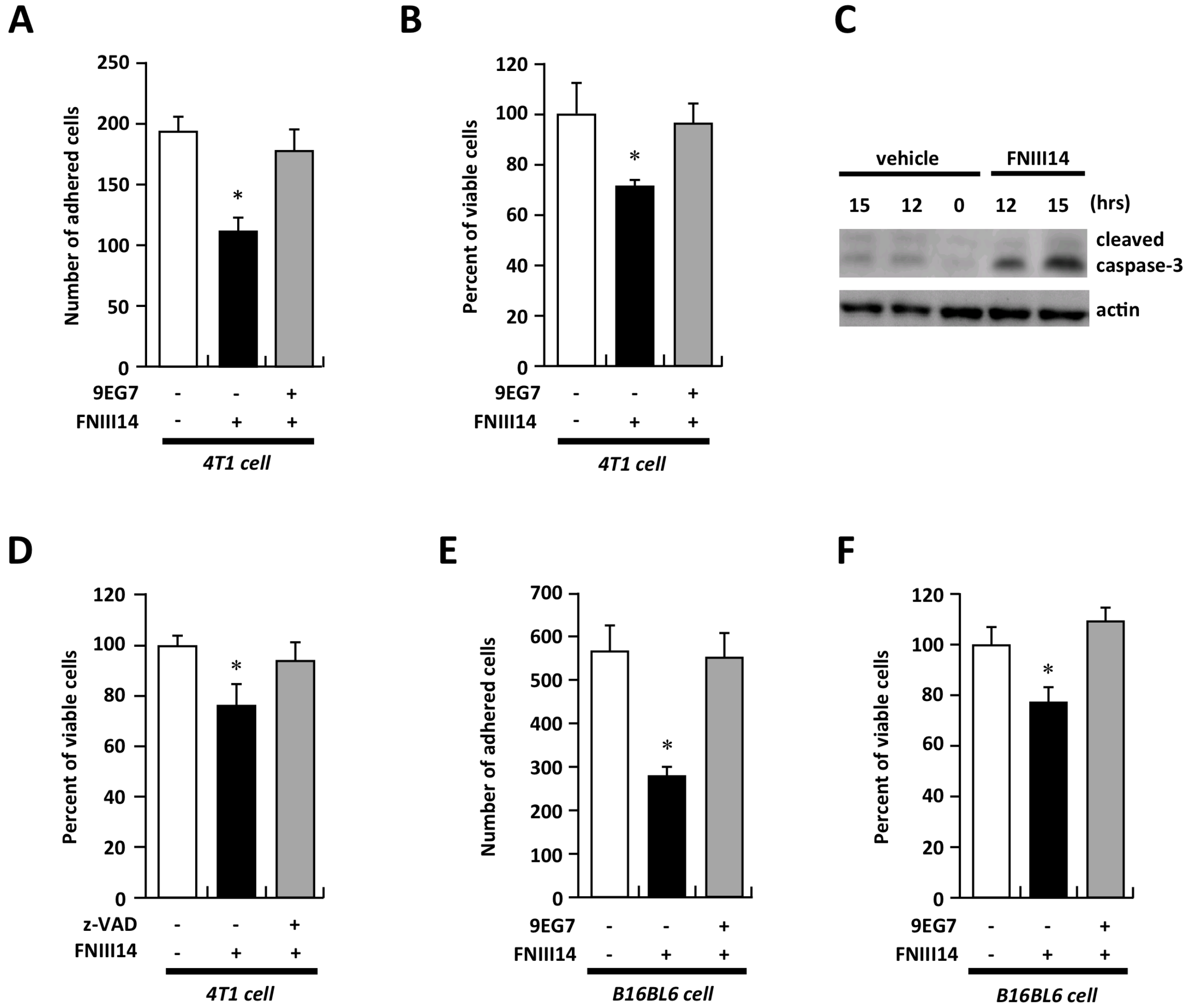

S1 Figure. Adhesion and survival of tumor cells treated with anti-adhesive peptide FNIII14. 4T1 (A, B, D) or B16BL6 (E, F) cells were seeded on fibronectin-coated plate with/without peptide FNIII14, β1 integrin activating antibody 9EG7, and caspase inhibitor Z-VAD. One and a half hours later, number of adhered cells (both spread and attached cells) in random 5 fields was counted after crystal violet staining (A, E). Twenty-four hours later, the number of viable cell was estimated by WST assay (B, D, F). Data were shown as means ± S.D.. *; $p<0.05$ vs FNIII14(-)/9EG7(-) cells. (C) Cleavage of caspase-3 in MMT cells with FNIII14 treatment was evaluated by western blotting.
