# Supplementary material for: Coadministration of the FNIII14 Peptide Synergistically Augments the Anti-Cancer Activity of Chemotherapeutic Drugs by Activating Pro-Apoptotic Bim
Source: PLoS One. 2016 Sep 13;11(9):e0162525. doi: 10.1371/journal.pone.0162525 (PMC5021278; doi:10.1371/journal.pone.0162525)
Supplement: S2 Fig — (A) Doxorubicin (DOX)-treated 4T1 cells with/without FNIII14 administration was harvested and intracellular fluorescence was measured by flowcytometer. (B) FITC-conjugated vinblastine (VBL)-treated B16BL6 cells with/without FNIII14 administration was harvested and intracellular fluorescence was measured by flowcytometer. (PDF) [file pone.0162525.s003.pdf]

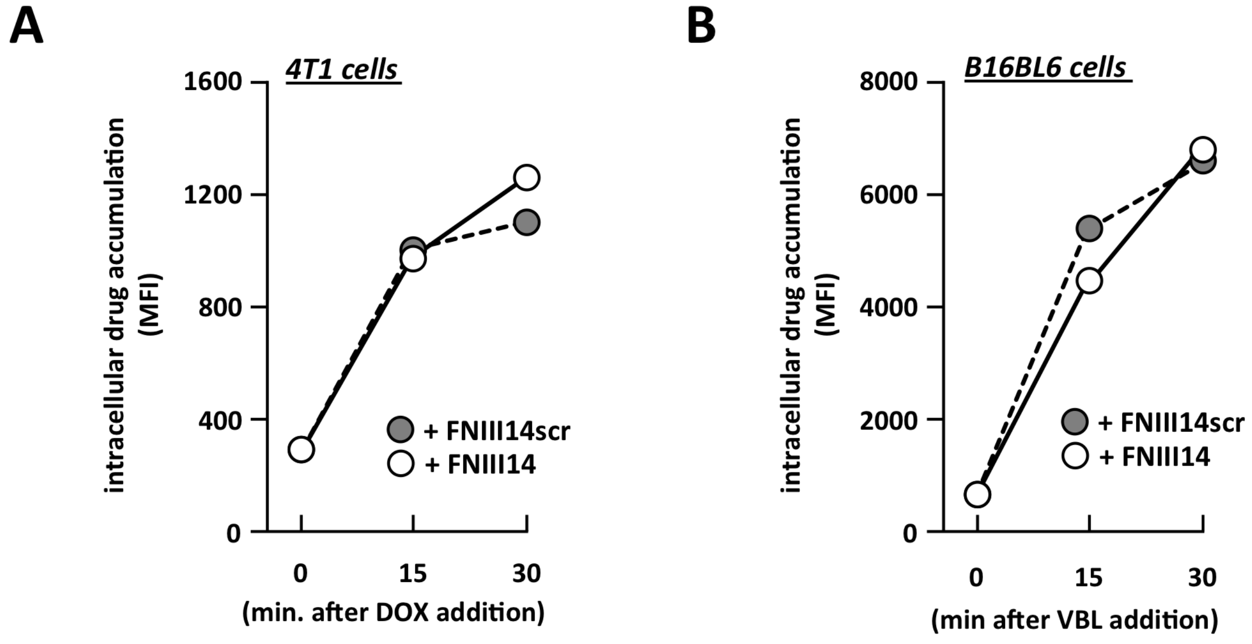

S2 Figure. E**ffect of FNIII14 on intracellular accumulation of chemotherapeutic drug.**

(A) Doxorubicin (DOX)-treated 4T1 cells with/without FNIII14 administration was harvested and intracellular fluorescence was measured by flowcytometer. (B) FITC-conjugated vinblastine (VBL)-treated B16BL6 cells with/without FNIII14 administration was harvested and intracellular fluorescence was measured by flowcytometer.
