# Supplementary material for: Coadministration of the FNIII14 Peptide Synergistically Augments the Anti-Cancer Activity of Chemotherapeutic Drugs by Activating Pro-Apoptotic Bim
Source: PLoS One. 2016 Sep 13;11(9):e0162525. doi: 10.1371/journal.pone.0162525 (PMC5021278; doi:10.1371/journal.pone.0162525)
Supplement: S3 Fig — (A) 4T1 cells, stimulated with peptide FNIII14, was stained using anti-Bim (red) and anti-tubulin (green) Abs. Representative images of confocal microscopic analysis from three independent experiments are shown. (B) Traces of fluorescence intensity spatial profile through the white broken line shown in confocal images (upper left of each histogram). White arrowhead displays a positive correlative colocalization of Bim and tubulin, or Bim and mitochondria, while white arrow indicates a absence of correlative colocalization. (PDF) [file pone.0162525.s004.pdf]

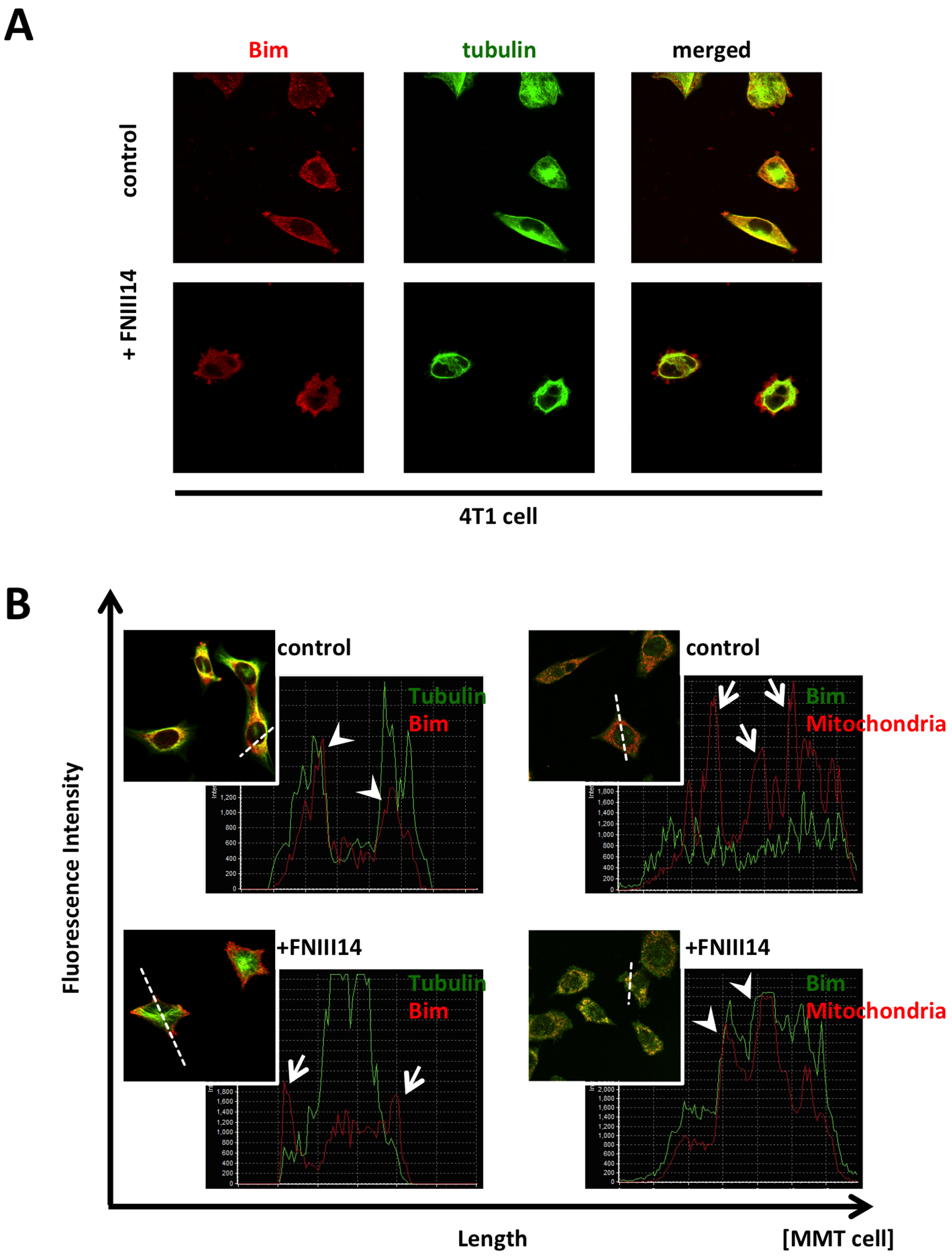
A
Bim
tubulin
merged
control
+ FNIII14
4T1 cell
B
control
Tubulin
Bim
control
Bim
Mitochondria
+FNIII14
Tubulin
Bim
+FNIII14
Bim
Mitochondria
Fluorescence Intensity
Length
[MMT cell]

S3 Figure. **Activation of Bim signaling pathway in mammary tumor cells with FNIII14 treatment.**

(A) 4T1 cells, stimulated with peptide FNIII14, was stained using anti-Bim (red) and anti-tubulin (green) Abs. Representative images of confocal microscopic analysis from three independent experiments are shown. (B) Traces of fluorescence intensity spatial profile through the white broken line shown in confocal images (upper left of each histogram). White arrowhead displays a positive correlative colocalization of Bim and tubulin, or Bim and mitochondria, while white arrow indicates a absence of correlative colocalization.
