# Supplementary material for: Coadministration of the FNIII14 Peptide Synergistically Augments the Anti-Cancer Activity of Chemotherapeutic Drugs by Activating Pro-Apoptotic Bim
Source: PLoS One. 2016 Sep 13;11(9):e0162525. doi: 10.1371/journal.pone.0162525 (PMC5021278; doi:10.1371/journal.pone.0162525)
Supplement: S4 Fig — Body weight (A), implanted primary tumor size (B), and the weight of lung (C) and spleen (D) at day 12 after tumor graft implantation was shown in bar graph. Data were shown as means ± S.D. *; p<0.05 vs Dox(-)/FNIII14(-) animals. (PDF) [file pone.0162525.s005.pdf]

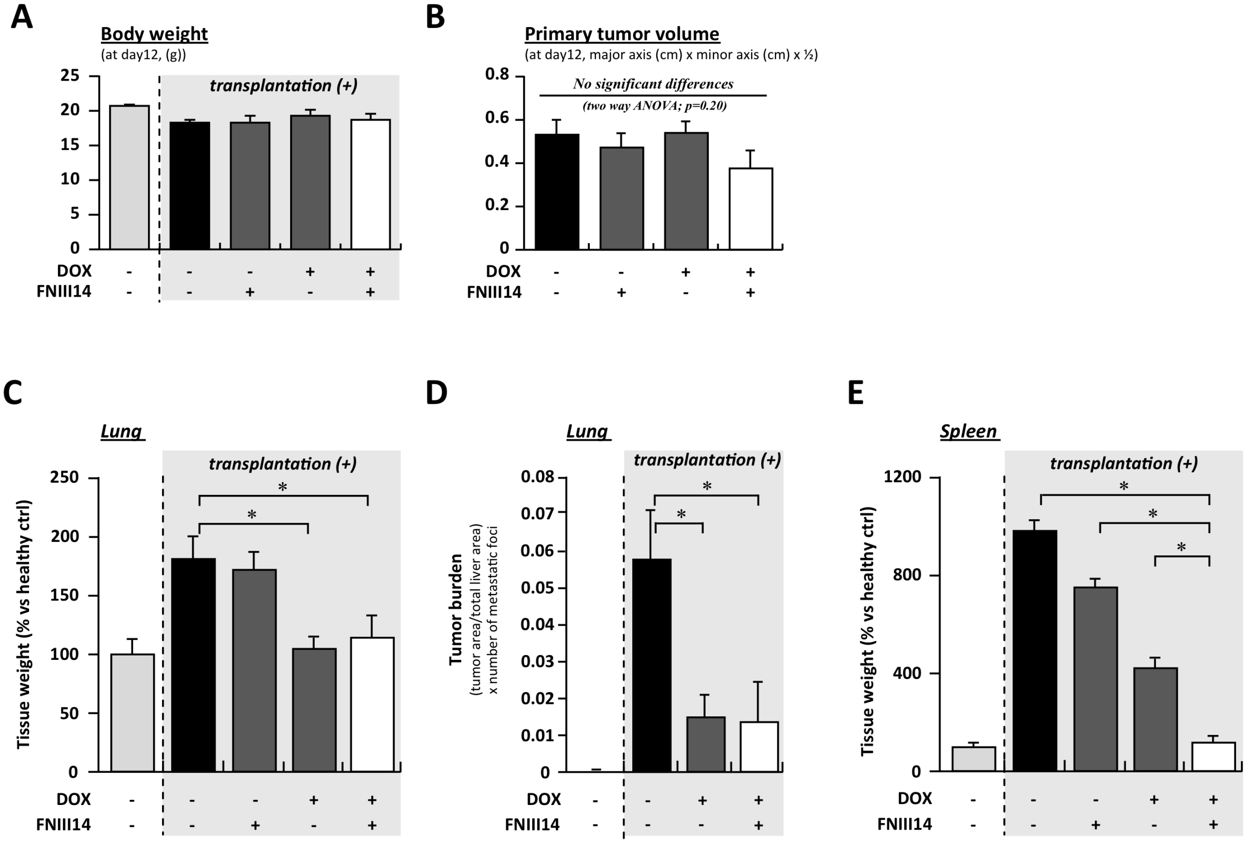

S4 Figure. Combination therapy using FNIII14 and doxorubicin in mouse mammary tumor metastasis model.

Body weight (A), implanted primary tumor size (B), and the weight of lung (C) and spleen (D) at day 12 after tumor graft implantation was shown in bar graph. Data were shown as means ± S.D.. *; p<0.05 vs Dox(-)/FNIII14(-) animals.
